# Risk of secondary malignant neoplasms in children following proton therapy vs. photon therapy for primary CNS tumors: A systematic review and meta-analysis

**DOI:** 10.3389/fonc.2022.893855

**Published:** 2022-08-12

**Authors:** Rituraj Upadhyay, Divya Yadav, Bhanu P. Venkatesulu, Raj Singh, Sujith Baliga, Raju R. Raval, Margot A. Lazow, Ralph Salloum, Maryam Fouladi, Elaine R. Mardis, Nicholas G. Zaorsky, Daniel M. Trifiletti, Arnold C. Paulino, Joshua D. Palmer

**Affiliations:** ^1^ Department of Radiation Oncology, The James Comprehensive Cancer Center, Ohio State University, Columbus, OH, United States; ^2^ Department of Radiation Oncology, The University of Texas MD Anderson Cancer Center, Houston, TX, United States; ^3^ Department of Radiation Oncology, Loyola University, Chicago, IL, United States; ^4^ Department of Radiation Oncology, Virginia Commonwealth University, Richmond, VA, United States; ^5^ Department of Pediatrics, Nationwide Children’s Hospital, Columbus, OH, United States; ^6^ Department of Radiation Oncology, University Hospitals Seidman Cancer Center, Case Western Reserve School of Medicine, Cleveland, OH, United States; ^7^ Department of Radiation Oncology, Mayo Clinic, Jacksonville, FL, United States

**Keywords:** secondary Malignant Neoplasms after proton therapy vs photon therapy secondary cancer, proton therapy, CNS radiation, pediatric cancer, photon

## Abstract

**Background:**

Central nervous system tumors are now the most common primary neoplasms seen in children, and radiation therapy is a key component in management. Secondary malignant neoplasms (SMNs) are rare, but dreaded complications. Proton beam therapy (PBT) can potentially minimize the risk of SMNs compared to conventional photon radiation therapy (RT), and multiple recent studies with mature data have reported the risk of SMNs after PBT. We performed this systematic review and meta-analysis to characterize and compare the incidence of SMNs after proton and photon-based radiation for pediatric CNS tumors.

**Methods:**

A systematic search of literature on electronic (PubMed, Cochrane Central, and Embase) databases was conducted in accordance with the Preferred Reporting Items for Systematic Reviews and Meta-Analyses (PRISMA) method. We included studies reporting the incidence and nature of SMNs in pediatric patients with primary CNS tumors. The crude incidence of SMNs and all secondary neoplasms were separately extracted, and the random-effects model was used for pooled analysis and subgroup comparison was performed between studies using photons vs. protons.

**Results:**

Twenty-four studies were included for analysis. A total of 418 SMNs were seen in 38,163 patients. The most common SMN were gliomas (40.6%) followed by meningiomas (38.7%), sarcomas (4.8%), and thyroid cancers (4.2%). The median follow-up was 8.8 years [3.3–23.2].The median latency to SMN for photons and protons were 11.9 years [5-23] and 5.9 years [5-6.7], respectively. The pooled incidence of SMNs was 1.8% (95% CI: 1.1%–2.6%, I^2^ = 94%) with photons and 1.5% (95% CI: 0%–4.5%, I^2^ = 81%) with protons. The pooled incidence of all SNs was not different [photons: 3.6% (95% CI: 2.5%–4.8%, I^2^ = 96%) vs. protons: 1.5% (95% CI: 0–4.5%, I^2^ = 80%); p = 0.21].

**Conclusion:**

We observed similar rates of SMN with PBT at 1.5% compared to 1.8% with photon-based RT for pediatric CNS tumors. We observed a shorter latency to SMN with PBT compared to RT. With increasing use of pencil beam scanning PBT and VMAT, further studies are warranted to evaluate the risk of secondary cancers in patients treated with these newer modalities.

## Introduction

Central nervous system (CNS) tumors are now the most common neoplasms seen in children and adolescents, comprising about one-fourth of all childhood cancers, and remain the leading cause of cancer-related deaths in this population (1–3). Of these, the most common tumor types seen are glioma, medulloblastoma, and germ cell tumors. Radiation therapy (RT) is a key component in multimodal management of most of these tumors. Although survival outcomes in children have improved with advances in radiation techniques, treatment-related late effects continue to be a concern. Secondary malignant neoplasms (SMNs) are one of the most dreaded complications, particularly in the pediatric population given significantly higher rates of SMNs noted than in adults following RT (4). The cumulative risk of secondary malignant brain tumors after therapeutic photon-based cranial irradiation has been reported to be 0.5%–3.7% at 10–15 years (5, 6). Recent strategies to decrease the integral dose and subsequently minimize late toxicities in the pediatric population include the use of more conformal RT techniques such as intensity-modulated radiation therapy (IMRT). However, even with more targeted photon-based therapy, higher integral doses due to more monitor units, leakage radiation, and higher volume of tissues exposed to low-dose radiation can result in more SMNs (7).

An emerging area of interest to minimize the risk of SMNs after RT is proton beam therapy (PBT). The lack of an exit dose with proton therapy due to the Bragg peak phenomenon may reduce the risk of SMNs in the pediatric population (8). This is especially useful in patients with medulloblastoma who often receive craniospinal irradiation leading to increased exposure of thoracic and abdominopelvic structures to RT. Although protons theoretically should be associated with a lower risk of SMNs, secondary neutrons resulting from various apparatuses along the proton beamline as well as the radiobiologic uncertainties have been concerning (9). Secondary cancers commonly occur >5–10 years after RT (10). The widespread use of proton therapy in the United States started at the beginning of the 21st century and only recently have mature data been available with adequate follow-up to estimate late effects of protons. Hence, there are limited data comparing the incidence of SMNs following photon based RT vs. PBT.

It becomes difficult to establish the true incidence of SMN due to the fact that in addition to radiation exposure, the genetic abnormalities (e.g., Li-Fraumeni syndrome) and risk factors associated with primary tumors (e.g., smoking) could predispose the individuals to develop a second cancer. Given that radiation is often the standard of care or an important salvage treatment for most CNS tumors, it becomes challenging to have randomized or matched cohorts comparing the risk of secondary cancers in irradiated and non-irradiated patients. Thus, we undertook this systematic review to evaluate the current literature on secondary cancers and to characterize and compare the incidence of SMNs after proton and photon-based radiation for pediatric CNS tumors.

## Methods

A systematic search of literature was conducted according to the Preferred Reporting Items for Systematic Reviews and Meta-Analyses (PRISMA) method. **Supplementary Table 1** shows the PRISMA checklist. The PubMed (National Institutes of Health), Cochrane Central (Cochrane collaboration), and Embase (Elsevier) databases were queried with the Medical Subject Heading (MeSH) terms—”secondary malignant neoplasm or second primary or radiation-induced”, “radiotherapy or radiation or irradiation”, “child or pediatric or adolescent”, and “brain or nervous system neoplasm”. Appropriate synonyms were determined and searched for as text in article titles, abstracts, and keywords. The search did not have a language filter. After removing duplicates, a total of 1,168 articles were identified. RU and JP did the search independently and any disagreements were resolved by mutual discussion.

### Eligibility criteria for articles

The inclusion criteria were as follows: (i) any prospective, retrospective, or cohort study reporting the incidence and nature of SMNs in a cohort of at least 10 pediatric patients with primary CNS tumors; (ii) study should include patients with age <21 years; (iii) original article in English language or an available translated version.

The exclusion criteria were as follows: (i) review articles and isolated case reports; (ii) preclinical and modeling studies without clinical information; (iii) studies describing only benign secondary tumors such as meningiomas and pituitary adenomas, or secondary skin cancers alone; (iv) papers with incomplete, missing, or duplicated data. Studies reporting the incidence of SMN in a cohort inclusive of significant number of patients not receiving RT were excluded as well (11–14). We did not require a minimum median follow-up duration as most proton literature have less long-term follow-up data available.

### Article review

**Figure 1:**
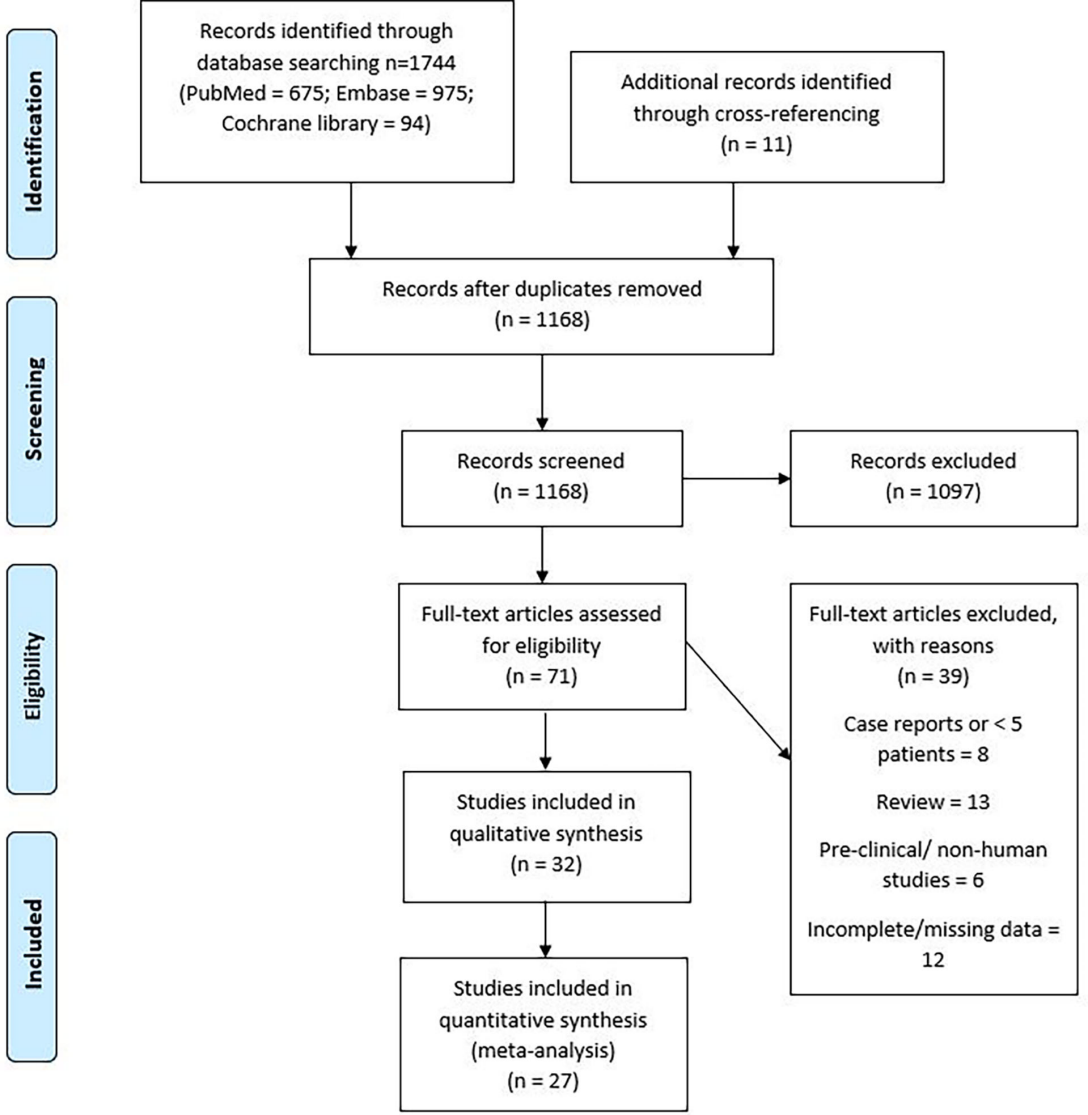
PRISMA flow diagram, depicting the search strategy.

The search process was performed consistent with the PRISMA flow diagram shown in **Figure 1**. The articles from the initial search were imported into Reference Manager Software. The duplicates were excluded, and the titles of articles were evaluated. The articles were independently reviewed by two authors (RU and JP), and relevant studies were identified. The articles found to be relevant to the topic of interest were shortlisted, and the full-length paper of these articles was assessed for the eligibility criteria. The included study references were cross-searched for additional studies. SMNs were defined based on Cahan’s criteria described by a classic study examining sarcomas developed in irradiated bone (15): (1) history of radiation exposure and presence of tumor in a previously irradiated region, (2) sufficient latency time between the original and new tumors, (3) histology of the new tumor must be distinct from the original that is typically seen as a second neoplasm, and (4) the tissue in which the alleged induced tumor arose must have been normal (i.e., metabolically and genetically) prior to the radiation exposure. For eligible studies, data were extracted as available, including the total number of patients, pediatric patients, radiation modality, median follow-up, incidence and cumulative incidence of SMNs, initial and secondary tumor types, median interval between RT and occurrence of SMNs, and the location of SMNs with respect to the radiation target. Secondary “malignant” neoplasms and all secondary neoplasms (SNs) were separately assessed and analyzed. SMNs included neoplasms like high-grade gliomas, sarcomas, thyroid cancers, and basal cell carcinoma, while benign neoplasms like meningioma, thyroid adenoma, osteoma, and desmoid tumors were included as SNs but not SMNs.

### Statistical analysis

Crude incidence of SMNs and all secondary malignancies were separately tabulated along with 95% confidence intervals, and the random-effects model described by DerSimonian and Laird was used for pooled analysis of incidence. The study-specific incidence rates were standardized to a cumulative incidence over 10 years for each study. The forest plot illustrating this information was plotted by the generic inverse variance method, the Jackson method for confidence intervals of tau and tau (2). Study heterogeneity was assessed using the inconsistency index (I^2^ statistic) with values of 0%–30%, 31%–60%, 61%–75%, and 76–100% indicating low, moderate, substantial, and considerable heterogeneity, respectively. A subgroup comparison was performed between studies using photons and protons. Meta-analyses were performed using R 3.6.1 (https://www.r-project.org/; Vienna, Austria), R package meta was used for meta-analysis, and statistical significance was set at p < 0.05 (16).

## Results

An electronic search of PubMed, Embase, and Cochrane Central identified 1,744 articles. **Figure 1** describes the PRISMA flow diagram of the search strategy. A cross-reference search of included studies further identified 11 new studies. After removal of duplicates, 1,168 abstracts were assessed. After assessing the title and/or abstracts, 71 articles were found to be relevant to the research question. The full-length articles of these studies were assessed, and 24 studies were found to fit the inclusion criteria (5, 16–38). Notable exclusions were case reports, review articles, and studies in preclinical models. Studies reporting the incidence of SMNs in a non-pediatric population were also reviewed for systematic review but not for meta-analysis. In studies reporting SMNs in all age groups and other tumor types, data for only pediatric patients with primary CNS tumors were extracted from the available segregated data. The studies with possible overlap were also excluded. Among the included studies, there was a potential overlap between patient populations only between two studies by Neglia et al. (2006) and Armstrong et al. (2009), who reported second cancers on the St. Jude’s Childhood Cancer Survivor Study (CCSS). The Newcastle–Ottawa scale assessment was used to assess the quality of the studies included in the systematic review and is presented in the supplementary data (**Supplementary Table 2**).

A total of 418 SMNs and 645 any secondary neoplasms were seen in 38,163 patients overall. The most common secondary neoplasms seen were gliomas (40.6%) followed by meningioma (38.7%), sarcomas (4.8%), thyroid cancer (4.2%), and basal cell carcinoma (1.3%) (**Table 1**). The overall median follow-up was 8.8 years (range: 3.3 to 23.2 years), while the median latency to a secondary cancer was 9.8 years (range: 5 to 23 years). Twenty-one studies (n = 36,763) reported the incidence of SMNs with photons (6, 17–36), while four studies reported the incidence of SMNs after protons (36–39) (n = 1,400). **Table 1** summarizes the details of these studies. The median follow-up for photons and protons was 8.8 years (range: 3.5 to 23.2 years) and 6.9 years (range: 3.3 to 12.8 years), respectively, while the median latency to a secondary cancer was 11.9 years (range: 5 to 23 years) and 5.9 years (range: 5 to 6.7 years), respectively.

**Table 1:** Summary of studies included in the study (PICOS).

S no.	Study author/year (Location/database)	Study design	N (CNS tumors)	No. of SMNs	No. of all SNs	Cumulative Incidence*	Median f/up (years)	Median latency (years)	Histology	Comments
Photon										
1	Stavrou 2001 (17) (Washington)	Retrospective	82	4	4	NR	7.6	7.0	Meningioma = 1, glioblastoma = 1, BCCs = 2	Medulloblastoma only, 3 of 4 patients were <3 years of age
2	Gold 2003 (18) (Minnesota)	Retrospective	79	3	7	3.4%	19.5	18.0	Meningioma = 4, astrocytoma = 1, BCC = 1, thyroid = 1	ALL and female gender at significantly higher risk of developing SMN. Eleven of 160 ALL patients (receiving cranial RT) developed SNs
3	Paulino 2004 (19) (UIowa)	Retrospective	429	13	23	NR	23.2	10.1	BCC (spine) = 1, sarcoma = 1, colonic adenocarcinoma = 1; benign adenomas = 3	71% were at the edge or inside the RT field
4	Broniscer 2004 (20) (SJCRH)	Retrospective	764	15	21	1.4%	3.5	7.9	Meningioma = 5, gliomas = 10, MDS = 1, BCC = 2, desmoid = 1, MFH = 1, ALL = 1	Age ≤2 years was a significant risk factor, not after exclusion of genetically predisposed (n= 7). 10-year CI of 4.4% for medulloblastoma and 20.2% for choroid plexus tumors
5	Neglia 2006 (21) (CCSS)	Prospective	14, 361	50	116	NR	NR	5.0	Gliomas = 40, meningiomas = 66, PNET = 6, CNS lymphoma = 1	For glioma, the risk was highest among children exposed at age <5 years. Gliomas at 9 years and meningiomas at 17 years after RT.
6	Hoff 2009 (22) (Germany)	Prospective	280	9	12	NR	8.8	8.0	HGG = 4, meningioma = 2, thyroid Ca = 2, sarcoma, melanoma, colonic adenocarcinoma, jaw osteoma	Medulloblastoma only
7	Merchant 2009 (23) (SJCRH)	Prospective	153	3	4	7 years = 2.3%	5.3	5.2	HGG = 2, LGG = 1, papillary thyroid Ca = 1	Ependymomas only, all SMNs in females, with age <4 years.
8	Armstrong 2009 (24) (CCSS)	Prospective	1, 085	16	20	25 years = 4.5%	15.4	14.0	Gliomas = 15, meningiomas = 4, PNET = 1	25-year CI of 7.1% in patients receiving cranial RT ≥50 Gy; 5.1% with RT <50 Gy and 1% with no RT.
9	Taylor 2010 (25) (Britain)	Retrospective	9, 223	57	57	40 years = 3.6%	17.3	20.5	All gliomas	Increased risk with RT >30 Gy and intrathecal methotrexate
10	Vinchon 2011 (26) (France)	Prospective	552	3	34	8.9%	6.9	13.1	Meningiomas = 26, gliomas = 2, meningosarcoma = 1, thyroid tumors = 6	Cavernomas were included in calculating CI.
11	Galloway 2012 (27) (UFlorida)	Retrospective	370	6	16	NR	22.4	18.9	Meningioma = 10, glioma = 4, sarcoma = 1, thyroid = 1	12.5% in boost volume, 25% marginal to the target volume, 6% distant to the target volume.
12	Packer 2013 (28) (Washington)	Prospective	379	13	15	4.2%	8.9	5.8	HGG = 6, LGG = 1, BCC = 1, sarcoma = 2, ALL = 1, MDS = 2, thyroid = 2	Medulloblastoma only. No significant difference in the incidence at age <5 years.
13	You 2013 (29) (Korea)	Retrospective	558	6	7	NR	10.9	9.5	Meningioma = 1, HGG = 4, LGG = 1, medulloblastoma = 1	All patients developing SMN received CSI.
14	Harbron 2014 (30) (Britain)	Retrospective	3, 150	27	32	NR	7.6	NR	Meningioma = 4, glioma = 4, Schwannoma = 1, sarcoma = 8, leukemia = 6, others = 9	68% in-field or within 8 cm of field edge
15	Christopherson 2014 (31) (UFlorida)	Retrospective	53	1	4	NR	15.4	11.9	Meningioma = 3, GBM = 1, RMS = 1	Medulloblastoma only
16	Tsui 2015 (32) (SJCRH)	Retrospective	2, 102	49	63	3%	10.3	NR	Meningiomas = 13, gliomas = 23, thyroid = 8, Schwannoma = 1	
17	Lee 2018 (33) (Taiwan)	Retrospective	681	14	27	25 years = 3.96%	NR	14.6	Meningiomas = 13, sarcomas = 7, and HGG = 6	Age <7 years and CSI were significant predictors
18	Bavle 2018 (6)	Retrospective	55	3	6	3.7%	8.1	NR	NR	Part of a pooled meta-analysis
19	Remes 2019 (34) (Finland)	Retrospective	73	0	6	0%	19.9	23.0	Meningiomas = 6, Schwannoma =1	No SMNs seen
20	Nantavithya, 2020 (35) (SEER)	Retrospective	2,271	104	146	3.1%	NR	12.5	Meningioma = 42, glioma = 17	Medulloblastoma only, MC site CNS
Proton										
1	Chung, 2013 (37) (MGH)	Retrospective	249	10	10	5.4%	6.7	5.0	HGG = 4, Schwannoma = 2, others/unknown = 4	All ages. 10-year CI of SMNs in a matched photon cohort from SEER was significantly higher at 8.6% (HR 0.52).
2	Yock, 2016 (38) (MGH)	Prospective	59	0	0	0%	7.0	NA	NA	Medulloblastoma only. Lower dose CSI used in majority of the patients
3	Indelicato 2021 (39) (Uflorida)	Retrospective	1,040	6	7	5 years = 0.8% 10 years = 3.1%	3.3	6.7	HGG= 3, sarcoma = 2, Schwannoma = 1, LGG = 1	Higher risk in patients with tumor predisposition syndromes. 2 patients developed leukemias.
Mixed										
1	Paulino 2021 (36) (MD Anderson)	Retrospective	115 (Ph = 63, PBT = 52)	6 Ph : 4 PBT : 2	8 Ph:6 PBT:2	Photon: (5 years = 0%, 10 years = 8%) Proton: (5 years = 2.2% 10 years = 4.9%)	12.8	5.9	HGG = 2, sarcomas = 1, papillary thyroid cancer = 1, salivary gland cancer = 1, testicular GCT = 1, thyroid adenoma = 2	Medulloblastomas CSI only. SMNs after PBT occurred earlier at 32.6–65.9 months than after photon-based RT (75–144 months)

*Cumulative incidence of SMNs at 10 years unless specified. CNS, central nervous system; SMN, secondary malignant neoplasm; SN, secondary neoplasm; NR, not reported; SJCRH, St. Jude’s Children’s Research Hospital; CCSS, Childhood Cancer Survivor Study; SEER, Surveillance, Epidemiology, and End Results Program; MGH, Massachusetts General Hospital; NCDB, National Cancer Database; Ph, photon-based radiation; PBT, proton beam therapy; BCC, basal cell carcinoma; ALL, acute lymphoblastic leukemia; HGG, high-grade glioma; LGG, low grade glioma; MDS, myelodysplastic syndrome; MFH, malignant fibrous histiocytoma; PNET, primitive neuro-ectodermal tumor; RMS, rhabdomyosarcoma; GCT, germ cell tumor; CSI, craniospinal irradiation; CI, cumulative incidence.

**Figure 2:**
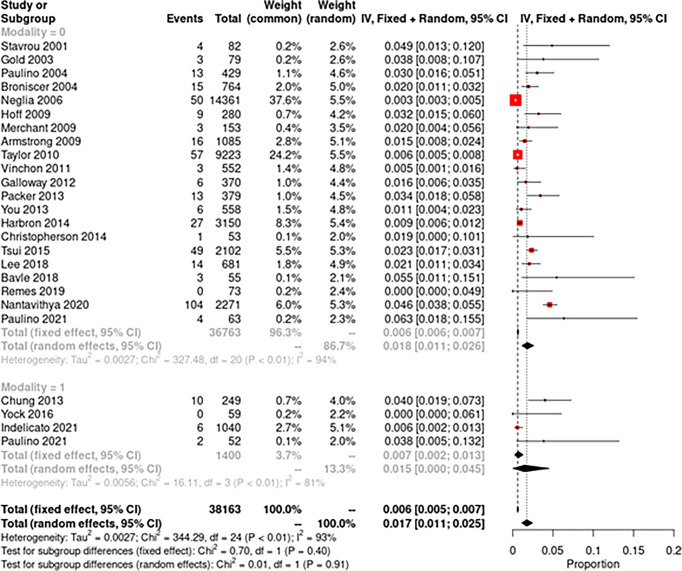
Forest plot demonstrating pooled analysis of secondary malignant neoplasms with photons (modality = 0) and protons (modality = 1).

**Figure 3:**
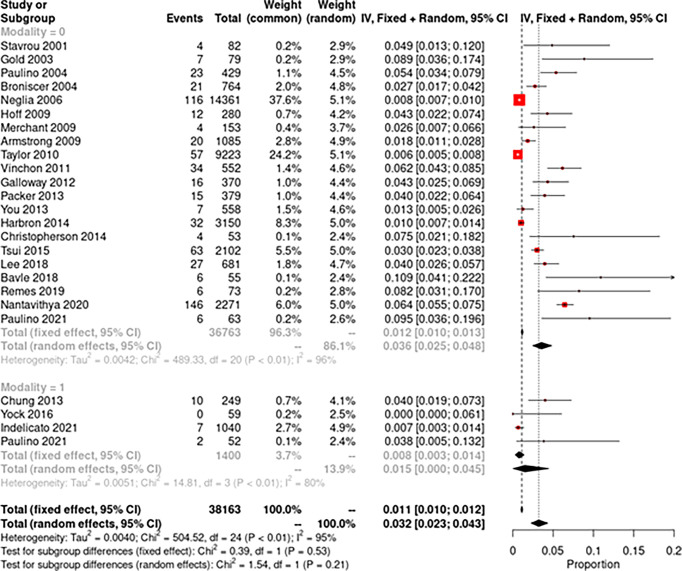
Forest plot demonstrating pooled analysis of all secondary neoplasms with photons (modality = 0) and protons (modality = 1).

The pooled random-effects incidence of SMNs with photons was 1.8% (95% CI: 1.1%–2.6%, I^2^ = 94%) while with protons it was 1.5% (95% CI: 0%–4.5%, I^2^ = 81%) (**Figure 2**). There was no significant difference among the two subgroups (p = 0.91). The pooled random-effects incidence of all secondary malignancies was also slightly lower with protons compared to photons, but the difference was not statistically different among the two groups [photons: 3.6% (95% CI: 2.5%–4.8%, I^2^ = 96%) vs. protons: 1.5% (95% CI: 0–4.5%, I^2^ = 80%); p = 0.21] (**Figure 3**). **Supplementary Figure 1** shows the funnel plots demonstrating publication bias for the respective forest plots.

### Incidence of SMNs with photon therapy

Early studies described radiation-induced neoplasms with low-dose RT in children who received RT for tinea capitis (mean dose 1.5 Gy) (40, 41). Meningiomas were the most common tumors seen with a relative risk (RR) of 9.5 compared to the general population, while that for glioma was 2.6. More recently, Packer et al. assessed the incidence of secondary tumors in 379 children with medulloblastoma treated per the COG-A9961 trial and estimated a 10-year cumulative incidence of 4.2% for SMNs (28). Bavle et al. found a 10-year cumulative incidence of 6.1% for all secondary neoplasms, 3.1% for secondary benign neoplasms, and 3.7% for SMNs in a retrospective study and meta-analysis including 1,114 patients (6). Similar studies in ependymoma survivors have shown a 7-year cumulative incidence of 2.3% (23), and those in germinoma survivors have shown an 11-year cumulative incidence of 9% (42). However, in the latter study, only one out of five second cancers were malignant, while the other four were benign meningiomas.

### Incidence of SMNs with proton therapy

Indelicato et al. assessed the risk of SMNs in 1,713 pediatric patients (1,040 CNS tumors) treated with passive scatter proton therapy and found a 5- and 10-year cumulative incidence of SMNs to be 0.8% and 3.1%, respectively. Paulino et al. (36) analyzed 115 children with medulloblastomas <18 years of age who received craniospinal irradiation with either photon craniospinal irradiation (CSI) and an intensity-modulated RT boost (n = 63) or passively scattered proton CSI and a boost (n = 52). They observed a 5- and 10-year risk of SMN to be 1% and 6.9%, respectively, without a difference in incidence by RT group (p = 0.74). There was no difference in the distribution of SMNs according to sex, age at RT (≤7 or >7 years), risk category, CSI dose (18.0–23.4 vs. 30.6–39.6 Gy), RT modality, or type of chemotherapy (SJMB vs. COG and other). Interestingly, SMNs after PBT occurred earlier at 2.5–5.5 years after RT, while SMNs after photon therapy occurred later at 6–12 years after RT. Of note, two SMNs occurred in the clinical target volume in the brain (malignant glioneuronal tumor, glioblastoma), two occurred in the exit dose region from the photon spinal field (papillary thyroid cancer, cardiac tumor), one occurred in the entrance path of a proton beam (parotid mucoepidermoid cancer), and one occurred outside the radiation field (testicular germ cell tumor).

Chung et al. (37) have compared the incidence of second cancers in patients who underwent proton therapy for a variety of diagnoses with a population-based cohort of matched patients treated with photon radiation. They matched 558 proton patients with 558 photon patients from the Surveillance, Epidemiology and End Results registry; one-third of the patients evaluated had a primary tumor of the central nervous system. The median duration of follow-up/latency time to second malignancy was 6.7/6.0 years for proton patients and 6.0/4.75 years for photon patients. Of note, the patients in the proton cohort often received a portion of their treatment with photons. The 10-year cumulative incidence rates for second malignancies were 5.4% for proton patients and 8.6% for photon patients. After adjusting for age, sex, year of diagnosis, and primary site, proton therapy was not related with an increased risk of second tumor. Second malignancies were seen up to 23 years after proton therapy, again emphasizing the need for a longer follow-up to determine the true incidence.

## Discussion

In our meta-analysis of 24 studies, the pooled incidence of SMNs in childhood cancers treated with photon-based radiation techniques was 1.8%, compared to 1.5% with protons. The reported overall cumulative incidence of SMN at 10 years across studies ranged from 1.4% to 8.9% for photons, versus 0% to 5.4% with protons. We did not find a statistically significant difference between the two groups with respect to SMNs or any SNs. The median follow-up for studies reporting outcomes with proton therapy was slightly shorter at 6.9 years compared to 8.8 years with photons. Also, the secondary cancers seem to occur after a shorter latency after RT with protons, with a median of 5.9 years, compared to 11.9 years with photons. Similar results have been reported by Paulino et al., who compared the risk of secondary cancers after proton- or photon-based CSI for pediatric medulloblastoma patients (36). It has also been previously reported that radiation-induced large-vessel vasculopathy is observed earlier after proton therapy for pediatric CNS tumors at a median of 1.5 years than with photon therapy where the median time to development of vasculopathy is 5 years (43). One reason for this finding could be exposure to secondary neutrons with proton therapy (9). Neutrons have different radiobiological properties with a higher linear energy transfer and may cause earlier onset of late effects.

Several preclinical studies have modeled the risk of SMNs after proton therapy. Arvold et al. calculated second tumor rates in 10 patients with benign intracranial meningiomas treated with PBT at Massachusetts General Hospital based on dosimetric comparisons between proton RT and photon RT treatment plans. They observed that the relative risk of second tumors was significantly lower for PBT compared to photon plans (1.3 vs. 2.8 per 10,000 patients per year, p < 0.002) (44). The excess risk of an intracranial RT-associated second tumor was calculated using the method proposed by Schneider et al., based on the concept of organ equivalent dose (45). Several other modeling studies have demonstrated a similar reduced risk of second tumors after PBT compared to photon radiation (46, 47). In another modeling study, PBT decreased the expected incidence of radiation-induced secondary cancers for rhabdomyosarcoma by a factor of 2 and for medulloblastoma by a factor of 8 to 15 when compared to intensity-modulated RT and conventional photon-based RT, respectively (48). Despite these modeling studies suggesting dosimetric advantages of protons in preventing SMNs, an important caveat is that in order to achieve this benefit protons must be delivered by pencil beam scanning rather than passive scattering technique. The passive modulation proton technique can potentially expose the patient to an even higher dose of radiation distant from the target due to secondary neutron production from the scattering foil (7). Neutrons can lead to considerable contribution to the integral dose in particular, since neutrons have a large quality factor and thus even a small physical dose can result in considerable biological effects (49). This at least in part may negate the Bragg peak effect of PBT and balances the overall integral dose. This is a potential reason for similar rates of SMN seen in our study in the two groups. In contrast, active scanning sweeps a fine pencil beam through the target and fewer neutrons are produced in the patient itself.

One of the major risk factors for development of SMNs is younger age at the time of RT. Age at RT of <7 years and craniospinal irradiation significantly increased the risk of a secondary tumor (p <.05) in a cohort of 681 patients (33). Also in this study, secondary tumors developed in 11 of 128 patients (8.6%) with primary medulloblastomas, which was higher than the overall cumulative incidence (33). This is suggestive of the impact of radiation volume, which is significantly higher for medulloblastomas, on incidence of SMN as well. Another major factor associated with SMNs is genetic predisposition, for example Li-Fraumeni syndrome. A majority of patients developing basal cell carcinoma had underlying Gorlin syndrome or multiple-nevus syndrome. There is heterogeneity among several studies in defining SMNs, as some studies exclude genetically predisposed patients who develop SMNs while most others do not.

There is strong evidence that secondary tumors are more frequent in patients who receive both radiotherapy and chemotherapy (50). Although we excluded the studies reporting incidence of SMN in patients treated with chemotherapy alone, some of the secondary cancers seen in these studies especially hematological malignancies could be related to chemotherapy use, which was not separately analyzed, and could be one of the potential confounding factors on this study.

The natural history of the primary tumor affects the incidence of SBT; patients need to live sufficiently long to develop a SMN. The latency for benign neoplasms such as meningiomas is usually much longer than for malignant neoplasms such as high-grade glioma and sarcomas. The latency period for secondary tumors ranges from 5.5 to 30 years, with gliomas developing in 5–10 years and meningiomas developing around 15–20 years after radiation (51). Paulino et al. observed that male gender, cranial irradiation for leukemia, and use of craniospinal or whole-brain radiation were associated with a shorter latent time from RT to development of a meningioma, while patients receiving lower doses of RT had a longer latent time (51).

Among radiation-induced CNS neoplasms in adults, meningiomas are about 70%, gliomas about 20%, and sarcomas less than 10%. In children, the most common secondary neoplasm is malignant glioma, comprising around 40%–50% of all SMNs (52). We found a similar distribution in our study. For adult patients, a rough estimate of secondary malignancy of 0.1%–1% per decade after radiation is often quoted, extrapolated from long-term follow-up of patients treated for prostate (53) and cervical cancer (54) where surgical controls were compared to their radiation counterparts. Xiang et al. compared the risk of second cancers in 450,373 pediatric and adult patients who received 3D-CRT, IMRT, or PBT from the National Cancer Database (NCDB) (55). The overall incidence of SMN was 1.55 per 100 patient-years. There was no difference in the risk of second cancers between IMRT vs. 3D-CRT (adjusted OR = 1; 95% CI = 0.97–1.02), but PBT had an overall significantly lower risk compared to IMRT (adjusted OR = 0.31; 95% CI = 0.26–0.36). They also verified the consistency of results in propensity score-matched analyses, and overall, this study represents strong evidence suggesting a lower risk of SMNs with PBT, albeit with limitations of NCDB data completeness.

There are several limitations of this systematic review and meta-analysis, the most prominent of which is the high heterogeneity evident by the high I^2^ statistic. This is likely due to more and larger studies demonstrating the incidence of SMNs after photon therapy compared to studies reporting outcomes after PBT. Also, there is a heterogenous patient population across different studies, and heterogeneity in the treatment regimen with regard to chemotherapy, radiation modality, doses, and volumes. The overall follow-up with PBT is shorter than most of the photon cohorts, which may bias the true incidence of SMNs. Another major limitation is the different time periods of comparison. Assessment of SMNs often requires a long-term follow-up, which means our oldest study ranges from 1999 to 2021. The natural history of primary tumor can affect the incidence of SMNs as patients need to live sufficiently long enough to develop an SMN. In this context, improved survival with modern systemic drugs and surgical and RT techniques can impact the incidence of SMNs. In addition, among patients treated in recent eras of improved survivorship care including routine surveillance imaging, it is possible that this may lead to perhaps earlier or increased detection of SMNs. Finally, another important factor associated with SMNs is genetic predisposition. Nearly 10% children with pediatric cancers harbor cancer-predisposing genes (56), and this has not been clearly reported in these studies.

## Future recommendations

It has been conventionally suggested that secondary cancers often develop in tissues that receive a lower radiation dose or “low dose spill-off” receiving <2.5 Gy (11). This is also supported by the incidence of brain tumors after only diagnostic x-ray exposure (57). However, in a recent study by Galloway et al., the most common location of the second tumor was in the whole-brain field (57%) and in the moderate-dose range receiving 20–36 Gy (81%) (27). These findings suggest that along with reducing the low-dose area, decreasing the volume of brain receiving moderate radiation doses can substantially decrease the second tumor rates. This can be potentially accomplished by more conformal radiation techniques such as VMAT and PBS proton therapy. It has been predicted that IMRT can increase the risk of SMN due to more volume of irradiated tissue, especially low-dose bath and increased total monitor units delivered (7, 58), but clinical studies have not demonstrated a similar increased risk (55, 59). Also, limited studies have evaluated the use of volumetric arc modulated radiotherapy (VMAT), which is being used increasingly for medulloblastoma patients. VMAT is able to confine the CSI dose to the spine with less dose anteriorly in the thorax, abdomen, and pelvis. Treatment times are also faster compared with older methods of IMRT, with less leakage radiation expected, although there remains the disadvantage of a larger volume of normal tissue receiving low-dose RT. Similarly with proton therapy, the lower risk of SMNs is expected with pencil beam scanning proton therapy (PBS) and not passively scattered PBT because of an increased total body dose due to secondary neutrons in the latter (7, 60). A more frequent use of VMAT and PBS proton therapy, both of which decrease the irradiated tissue volume outside the clinical target volume, is recommended. In particular, for patients with medulloblastoma, treatment plans and protocols should focus on minimizing the amount of normal tissue receiving low to medium doses in the range of 20–36 Gy, by decreasing the craniospinal dose while keeping a posterior fossa boost dose similar. Continued avoidance of whole-brain RT for pediatric leukemia patients is recommended. Another approach to decrease low–medium-dose radiation exposure is to reduce the CTV margins and minimize PTV expansions with image guidance. In this regard, recent data from the ACNS0331 trial suggest changing the boost CTV volume from the entire posterior fossa to the tumor bed only and the ACNS 0831 study suggests decreasing boost margins in ependymoma without compromising local control and survival (61, 62). This aims to further decrease the risk of secondary cancers along with other normal tissue toxicities (63).

## Conclusion

Despite advances in radiation techniques, the risk of late secondary malignancies remains a concern, especially in the pediatric population. We observed similar rates of SMN with PBT at 1.5% compared to 1.8% with photon-based RT for pediatric CNS tumors in our meta-analysis of 24 studies, and the difference was not statistically significant. The risk of all secondary cancers was also lower, but the difference was not statistically significant. We observed a shorter latency to secondary cancers with proton therapy compared to photon-based radiation, which may be related to secondary neutron exposure. With increasing use of techniques that decrease the irradiated tissue volume outside the clinical target volume, like pencil beam scanning proton therapy and VMAT, further studies with a longer follow-up are warranted to evaluate the risk of secondary cancers in patients treated with these newer modalities.

## Data availability statement

The original contributions presented in the study are included in the article/**Supplementary Material**. Further inquiries can be directed to the corresponding author.

## Author contributions

RU, DY, BV and JP all conducted the literature search, data analysis. All authors performed data interpretation, manuscript writing and editing. All authors reviewed and accept the final manuscript.

## Conflict of interest

The authors declare that the research was conducted in the absence of any commercial or financial relationships that could be construed as a potential conflict of interest.

## Publisher’s note

All claims expressed in this article are solely those of the authors and do not necessarily represent those of their affiliated organizations, or those of the publisher, the editors and the reviewers. Any product that may be evaluated in this article, or claim that may be made by its manufacturer, is not guaranteed or endorsed by the publisher.
